# Graduates’ affective transfer of research skills and evidence based practice from university to employment in clinics

**DOI:** 10.1186/s12909-020-1988-x

**Published:** 2020-03-29

**Authors:** John Willison, Xiaoxin Zhu, Baolin Xie, Xuelin Yu, Jie Chen, Deng Zhang, Ishraga Shashoug, Fizza Sabir

**Affiliations:** 1 University of Adelaide, Adelaide, Australia; 2 Guangzhou Jiangnan Foreign Language School, Guangzhou, China; 3 Dongli Lake School, Tianjin, China; 4 Changsha Country Garden Venice Bilingual School, Changsha, China

**Keywords:** Cognitive and affective domains, Evidence-based practice, Professional degrees in health sciences, Qualitative research, Research skill development, Thematic analysis

## Abstract

**Background:**

This research sought to determine the impact of explicit program-based development of skills associated with research and Evidence Based Practice (EBP) on the attitudes and sustained behaviours of graduates subsequently employed in clinics. Systematic reviews have shown that university teaching of EBP and research skills rarely result in transfer of commensurate attitudes and sustained behaviours of students to their subsequent studies or to employment. Studies have therefore called for detailed exploration of what may enable this transfer of knowledge and skills to attitudes and behaviours. In keeping with these calls, this paper presents a fine-grained qualitative study of graduates’ research skills and EBP in clinics with particular reference to pertinent attitudes, values and behaviours sustained, or further developed, one year after program completion.

**Methods:**

The study revolved around employed graduates of a Bachelor of Oral Health (BOH) program, which used the Research Skill Development (RSD) framework to structure the explicit, coherent and cyclic development of the skills associated with research in multiple semesters of the degree. One year after their completion of the BOH program, semi-structured interviews were conducted with nine employed graduates, three from each of three consecutive cohorts, to gain their professional perspectives on their research skills and EBP developed at university and then used in clinics. While the pre-determined interview questions focused on employed graduates’ knowledge and skills, the attitudes and values around research skills and EBP emerged spontaneously.

**Results:**

Graduates that were interviewed relayed in detail their attitudes and values associated with research skills and EBP when asked about their work in clinics, even though the affective elements were not specifically elicited. In the employment context, the positive affective aspects of the skills associated with research and EBP that graduates discussed were pronounced, and this contrasted with working graduates retrospective view of university research skills and EBP.

**Conclusions:**

The richness of affective interaction with patients was a factor that enabled the interviewed graduates to transfer university knowledge and skills into attitudes and behaviours associated with EBP. We recommend similar fine-grained qualitative research to further develop constructs that enable quantification of the interplay of cognitive and affective facets in researching and EBP.

## Background

Health science programs strive to produce competent graduates who care for and about patients, are technically proficient, keep up with changes in knowledge and skills, and apply appropriate and reliable evidence to their practice. This is the drive to produce graduates who engage empathetically in Evidence-Based Practice (EBP). EBP has two main senses in the literature that may overlap or be in competition. EBP is most commonly conceived of as drawing on an existing evidence base that was produced by researchers and now used by clinicians [1]. The emphasis in this perspective is that the producers of the evidence base are distinct from clinicians and therefore the researchers have the epistemological power to recommend appropriate practice. This perspective sees clinicians as skilled consumers of published research, not with the capacity to generate original research [2].

A contrasting perspective to the above conceptualisation is that EBP requires more than application of researchers’ knowledge by clinicians. Rather, clinicians must be integrally involved in the process of knowledge generation, as well as context-sensitive determinations of how to apply the knowledge. This high-level involvement of clinicians is required because in EBP context-sensitive differences means that aspects that are effective in one situation may be counter-productive in another [3]. From that perspective, EBP practitioners also generate data and knowledge from their own practices to make content-sensitive decisions [4]. Because practitioners work in complex contexts, their workplaces require them to deal with barriers to the application of knowledge that are diverse, including those that are systemic in nature and those that are more affective, including motivational, emotional, and social barriers [4]. In this second perspective, the ‘clinician’ is more authoritative, and the ‘researcher’ provides useful information; the epistemological power to recommend appropriate practice is in the hands of the clinician.

These two perspectives of EBP may be considered to be in conflict or to be at either end of a continuum. In this paper EBP knowledge generation sometimes lies more along the researcher end of the continuum, and at other times more with the clinician, depending on the context, experience, cultural setting and patient. On this continuum, the skills and attitudes needed for EBP in clinical contexts are congruent with the cognitive skills and affective dispositions associated with research, where EBP and research skills are terms used at times synonymously and the processes are interdependent [2]. Both are, broadly, forms of active learning processes [5] with the focus of research on more generalizable findings and EBP on meeting patient needs.

Systematic reviews have consistently found a major issue for patient care is that knowledge and skills for research learning and EBP are substantially better developed in university programs than the associated attitudes and sustained behaviours of students and graduates [6–9]. Moreover, transfer of university-learned knowledge and skills to appropriate attitudes and sustained behaviours by graduates who work in clinical contexts is not only under-developed but also understudied [2].

While attitudes and values for EBP have been determined to be under-developed in a variety of articles, there are problems inherent with these quantitative studies. Many studies in health sciences have used self-report surveys to determine affective elements [10, 11] such as empathy [12, 13] and emotional intelligence [10] despite concerns with the reliability and validity of questionnaires that pertain to the affective domain [11]. For example, a longitudinal cohort study showed that empathy dropped in the 3rd year of a medical degree, and never recovered its earlier levels subsequently [12]. This may be a realistic measure of empathy, or the result may be a function of the instrument used. A literature review of 18 such studies that showed health science student empathy was stunted or dipped during their program concluded that affective aspects should be integral to clinical understanding, embedded across degrees not additional to them [14]. However, no studies sought to determine the development of student affective elements due to aspects that were integral to the whole degree program using measures more suited to affect. Moreover, while some studies do attempt to attribute to an intervention influence on student affective development, they suffer from being too far-removed from real-life patient interactions to deeply understand students’ empathy, once graduated and with patients under their care [13].

There is a need then to determine the extent of transfer of the knowledge and skills that students developed at university to the values, attitudes and sustained behaviours associated with research skills and EBP at their places of work well after graduation. This requires a deep, qualitative exploration to understand transfer of values, attitudes and behaviours to clinical workplaces [4] before further quantitative studies may be conducted. Therefore this article uses a fine-grained, context-sensitive phenomenological methodology to explore alignment of the knowledge and skills associated with researching and EBP that were explicitly developed in an allied health program, and the behaviours, values and attitudes of graduates of that program when they were employed in clinics.

This current research addresses the calls from quantitative researchers for fine-grained qualitative research [2] that deeply and richly explores context-sensitive connections between university teaching of EBP/research skills and sustained attitudes, values and behaviours of those who have graduated and now working in clinics. This study aimed to explore in depth the transfer of knowledge and skills to pertinent attitudes, values and sustained or further developed behaviours.

### The affective domain

In order to effectively deal with the issue of transfer of knowledge and skills to an individual’s attitudes and values, this affective dimension must first be clarified. The canonical Bloom’s Taxonomy of the Affective Domain [15] delineates the realm of affect in a hierarchy of five taxa that describe the extent of internalisation of values associated with learning: 1) *receiving* (the basic level), concerning awareness and willingness to pay attention to information; 2) *responding*, which involves willingness to actively engage in the learning process; 3) *valuing,* which is willingness to see the worth and significance of particular information; 4) *organizing,* which refers to the activities of harmonising different value systems and synthesising them; and 5) *characterizing*, which denotes ability to act consistently in the light of the values internalised, and to self-direct one’s actions in terms of these. In other words, the affective domain transcends liking or disliking; rather, it concerns processes which internalise learning, change behaviours and enable the move towards increasingly autonomous actions and attitudes that are consistent with one’s values [11].

In terms of student and graduate use of research skills and EBP, the top two levels of the Taxonomy of the Affective Domain are particularly important. ‘Organizing’ gives a sense of aligning various learning attitudes with values, and ‘Characterizing’ connotes the point at which sustained behaviours are in accord with these learning values. In effect, it is at these levels that people align what, when and how they learn and act with their values. At the top level, the habits of learning through research, seeking and probing evidence, may be considered more intuitive, ingrained, compelling, personal, instinctive or natural. However, ‘valuing’ (3rd Level) is an affective gatekeeper to these desirable top levels, and there is no way of skipping this level: it is not a step that may be jumped, but a precursor for and enabler of organisation and characterisation. But how can values that underpin a willingness to staying current with evolving knowledge and evidence be imparted to students?

### Conceptual framework on research skill development

In an Australian research university, the curriculum and assessment of a Bachelor of Oral Health was informed by the Research Skill Development (RSD) framework [16, 17], to promote and conceptually connect research skills and EBP. The RSD was implemented in 2008 in one course of the Bachelor of Oral Health, where in this pilot the marking rubric of one assessment was reframed using the RSD. By 2010, four courses in the first four semesters of the degree were using the RSD to frame assessment marking and to inform the learning process. An example of the RSD used as a rubric to guide the assessment in the BOH is available [18]. The final two semesters of the BOH incorporated a major clinical project in which research skills and EBP were required and for which students were assigned to a supervisor. In addition, students were given chances to work in the clinics (2–3 days per week) in the second and third years.

The RSD provided students and educators with guidance for the development of research skills [17] through the articulation of six facets of research elaborated along a five-level continuum of student autonomy. The RSD is a conceptual framework that is used by educators to facilitate student learning in a manner akin to researchers discovering and constructing knowledge, a process that feeds into, and is in parallel with, EBP as noted above.

The six facets of the RSD are:

#### Embark and clarify

Students commence research, responding to or posing questions and clarifying their needs for understanding, with an awareness of ethical, social and cultural issues. This includes identifying patient needs, defining problems and formulating working hypotheses.

#### Find and generate

Students use appropriate methodologies to find the information relevant to the topic and to generate the data needed, including taking patient history and recording presenting symptoms and signs.

#### Evaluate and reflect

Students critically evaluate the information found and the data collected and reflect on the processes used in research and clinical settings.

#### Organise and manage

Students organise the information found, and the data generated and manage individual and team processes and client assessments.

#### Analyse and synthesise

Students critically analyse the information and data and synthesise the knowledge created individually and in teams.

#### Communicate and apply

Students discuss, listen and respond, articulate processes used, and new knowledge developed, interact with audiences, such as patients or colleagues, and put new understanding into practice, heeding ethical, cultural and social issues [based on 16].

### The affective domain in research skill development

The RSD incorporates affective descriptors that map directly onto the cognitive facets mentioned above [16, 19]. Whereas Bloom, Krathwohl and colleagues devised separate taxonomies for cognitive [20] and affective [15] domains, the RSD combined these domains in the same framework [19]. While the cognitive facets of RSD are elaborated into a five-level continuum of autonomy [17], the affective facets are single-word adjectives that are suggestive, not prescriptive, of educational purpose, attitudes and values. Because the affective descriptors are co-located with the cognitive facets in the RSD, the former are imbued with meaning from the latter and vice versa, so that the two domains mutually reinforce [19]. Therefore, there is no separation of the cognitive and affective domains, which is in keeping with Bloom’s assertion [15] that ‘the fact that we attempt to analyse the affective area separately from the cognitive is not intended to suggest that there is a fundamental separation. There is none.’ Co-location in the RSD was vital, to overcome the dissonance associated with Bloom and Krathwahl’s statement versus their actual practice of devising two distinct taxonomies, which created an artificial separation of the cognitive and affective domains. In the RSD there is an acknowledgement of the differing emphases of the cognitive and affective domains and the framework represents them as co-existing on the same cognitive-affective spectrum of sophisticated thinking, learning, researching and EBP.

In terms of the affective domain, this current research looked for evidence in the interview transcripts of the values and attitudes associated with each cognitive facet of the RSD, which operate at the higher levels of the affective taxonomy to ‘characterise’ the graduates. For example, evaluative thinking is more on the cognitive side of the cognitive-affective spectrum, whereas *discerning* has a strong cachet, where it cuts through in a way that reflects values and attitudes and is considered more on the affective side of the spectrum in the RSD [19]. Einstein commented of his research process ‘I think and think for months, for years. Ninety-nine times the conclusion is false. The hundredth time I am right.’ [21]. Einstein’s willingness to discard error and not defend ideas once a flaw appeared shows that his values around the search for truth and beauty were at the top level of Bloom’s Affective Domain. For Einstein, being *discerning* involved a desire to sieve and reject until something worthy is found. In terms of Bloom’s affective domain, this is where values associated with evaluation characterise the employee as a *discerning* graduate who demonstrates this with sustained behaviours. Table 1 shows the published affective-oriented words associated with each RSD facet (16, 19).

**Table 1:** RSD facets and their associated affective descriptor (Willison and O’Regan, 2006/2018)

	RSD facet	Affective Descriptor
1	Embark and Clarify	Curious (Empathetic)
2	Find and Generate	Determined
3	Evaluate and Reflect	Discerning
4	Organise and Manage	Harmonising
5	Analyse and Synthesise	Creative
6	Communicate and Apply	Constructive

Each facet has an affective descriptor that operates at the higher levels of the Affective Domain taxonomy. To *embark & clarify* requires some motivating elements, which could be extrinsic or intrinsic. The values around embarking with a patient on their health journey, at the levels of ‘characterisation’, are epitomised by the adjectives *curious* for research, and *empathetic* for EBP [16, 19] and, for both, provide the motivation to embark on a process of finding out. A c*urious* graduate will be impelled by their own internalised values to instigate research to find out; an *empathetic* graduate will be compelled to care for a patient including using EBP to search for and negotiate optimal solutions.

In *finding & generating*, a graduate operating at the top levels of the Affective Taxonomy will not be slap-dash or cursory, but be *determined* and therefore have a willingness to keep going with processes appropriate to the context, resourcing and patient needs. The facet that seems most tedious to students, *organise & manage*, requires something impelling to drive values to be internalised and then expressed at the higher levels of the affective domain. A *harmonising* graduate is one who is impelled to *organise & manage*: Whether concerning information, data, process, teams, or timeframes, *the desire to be harmonising* is the driver of organising processes at the top level of the affective domain. For *analyse & synthesise*, the affective descriptor that sits in union with the facet is *creative*, and ‘create’ replaced synthesis in a revised cognitive Blooms Taxonomy [22]. Whereas *analyse & synthesise* sit most clearly far towards the cognitive end of the cognitive-affective spectrum, *creative* is better situated at the affective end in the RSD, and characterises the internalisation of values associated with that facet [19].

The final facet, *Communicate & Apply*, uses the adjective *constructive* in the way that the word is used in social contexts, such as ‘she’s a constructive person’ or ‘that was a constructive conversation’ [19]. These uses imply a disposition towards building-up, rather than, say, self-interest, and so is more on the affective side of the cognitive-affective continuum. *Constructive* as the RSD descriptor of higher levels of the Affective Domain may be contrasted with a standard educational use of the verb ‘to construct’, or nouns ‘construction’ and ‘constructivism’ which have more cognitive resonances.

While the cognitive aspects of the RSD have been well evaluated in a study spanning numerous disciplines and universities [23], no previous studies have tested these educator-conceived affective descriptors [19] with the realities of working graduates’ experiences of research and EBP in clinics.

## Aims and research questions

In a previous study of individual courses of the BOH program that used the Research Skill Development (RSD) framework to explicitly develop student research skills it was found that there were statistically and educationally significant positive changes in students’ perceptions of cognitive skill development when contrasted early to late in semester [23]. In contrast, no statistically significant changes were apparent in terms of the affective domain [23]. However, when students were interviewed one year after the RSD implementation, there were strong signs of affective elements, including that the research skills developed were perceived to be highly useful for employment [23]. Moreover, there was a risk, indicated in these interviews, that research skills may atrophy unless they were explicitly developed subsequently. Further research was called to determine ‘outcomes of explicit and coherent research skill development across entire degree programmes’ [23].

This current study sought to determine how the skills associated with research and EBP, that were explicitly developed in a three-year Bachelor of Oral Health (BOH) program, were perceived by graduates of the program in terms of their behaviour, values and attitudes now that they were employed in clinics, with patients under their duty of care. Therefore this current study first sought attribution by employed graduates of the influence of their whole BOH program on cognitive elements of their work with patients and colleagues. Then the research intended to find the breadth of affective descriptors that graduates ascribe, in day-to-day clinical contexts, to the more cognitive processes of the RSD facets. This affective-cognitive connection provides a deepened understanding of the issues associated with attitudes and sustained behaviours by graduates who work in clinical contexts [2].

The two research questions investigated were:

Research Question 1: What are graduates’ retrospective perspectives of how effectively the design features of the BOH degree explicitly developed student research skills and EBP?

This question allowed the capture of data that graduates attributed to the explicit *development* of research skill and EBP in the BOH program.

Research Question 2: One year after BOH degree completion, what are the affective descriptors that graduates associate with their research skill and EBP in clinical contexts?

Question 2 allowed the probing of how graduates intuitively connect the affective and cognitive elements of research skills and EBP when they are employed in clinics, and so provide insights into attitudes, values and sustained behaviours.

## Methods

In order to elicit employed graduates’ personal perspectives, this study employed a phenomenological methodology to provide optimum data to address the research questions [24, 25]. A phenomenological approach seeks for people’s experiential perspectives and prioritises participants’ social and psychological experiences [26, 27]. This approach was appropriate to capture the affective domain, which is by nature subjective and context-sensitive. This phenomenological perspective fostered rich description of each individual’s experiences of a shared phenomenon enabling deeper understanding of the phenomenon studied [24, 28], that is the graduates’ experience of the development of research skills and EBP in the BOH and of affective aspects of these skills as used in employment.

**Fig. 1:**
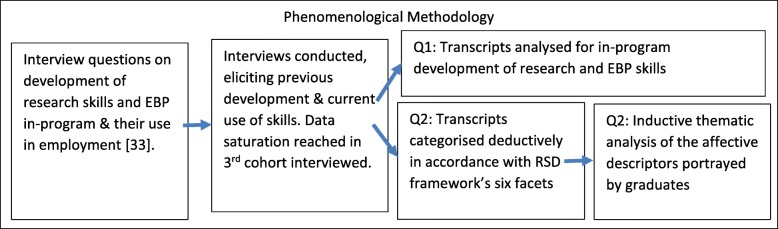
A flowchart of the processes in the methodology

Phenomenological research commonly uses semi-structured interviews [29] as a method of data collection [30] as they allow flexibility for participants to amplify their ideas and for the interviewer to seek further details [31]. The relationship between methodology, interview process, and data framing and analysis is shown in a flowchart of the study (Fig. 1).

### Data collection procedure

Graduates of the 2010, 2011 and 2012 cohorts of the three-year BOH program (with 35–40 graduates each year) were all invited by Facebook or enduring email to participate in interviews 1 year after completion of the program. No direct contact from the researchers was allowed by the ethics protocol, so emailed invitations from professional staff and social media invitations were used to make contact with graduates. Three participants from each of the 2010, 2011 and 2012 graduating classes volunteered to participate, resulting in a total of nine interviews, and providing a sample of graduates comprising only those willing to be interviewed. This 8–9% response rate is typical of long-term follow-up of interventions where direct contact is not permitted by ethics protocols [21]. The three interviews with the 2012 graduates, conducted in 2013, yielded now new themes, and it was deemed that data saturation had occurred [32], so no further interviews were necessary.

The semi-structured format of the interviews comprised a predetermined list of questions (see Appendix 1 for the prepared questions) which were developed in an earlier study [23], adapted and used in a broader study [33]. The interviewer fluidly adapted questions to further probe interviewees’ responses [29] which enabled the capture of rich details, in keeping with the semi-structure of the interviews. Interview questions elicited graduate retrospective perceptions of the development of research skills and EBP during the degree and their use during employment one year after completion of the degree. The intention behind this time-lag was that, due to their full year of employment, participants’ reflections would be more industry-relevant when compared to the perspectives of new graduates. During interviews, participants were not cued about the affective domain and these elements were rather allowed to emerge as unsolicited outcomes. Rather, when graduates provided specific accounts of interactions with patients this rich data sensitively reflected affective aspects of EBP.

The interviews were conducted face-to-face at a location of the participants’ convenience by a researcher experienced in semi-structured interviews. No researchers were involved in teaching the BOH program. All interviews were audio recorded, with each interview lasting between 40 and 60 min. Audio recordings were transcribed by a transcription service, and each recording resulted in a document of 15 to 20 pages.

### Data analysis procedure

Data analysis in this study comprised three phases where each graduate’s perspective on their use of research skills in their workplaces were categorised according to the six RSD facets, where the first two phases were deductive in nature and the third phase inductive. In the first phase, each of six coders worked on one allocated facet and determined their coding individually for one transcript at a time, highlighting the terms, phrases and sentences epitomising their facet of the RSD, whether cognitive or affective. Each coder then met with two other coders to review and give feedback about each other’s deductive coding. In addition, the research officer (who was experienced in coding RSD transcripts) coded all the six facets independently.

In the second, parallel, phase, the whole research team met weekly to discuss the coding of each transcript and these meetings were facilitated by the research supervisor. The purpose of these meetings was to decide whether the deductive coding of phrases and sentences for each facet was agreed upon by all. This procedure allowed each coder to review and discuss elements of transcripts that were coded differently or missed out, and the research officer to challenge coding that was different from her own. After each discussion, the data were reviewed again in the light of feedback from the team. This procedure was followed to enhance the credibility of the research, in accordance with other studies [34, 35], in which it was found that peer scrutiny and data rechecking enhanced the validity of analysis. The coding proceeded at one transcript per week for 5 weeks and then two per week for the final four transcripts.

The last, inductive, phase of analysis involved each researcher generating affective themes [36, 37] (called ‘descriptors’ below) for their allocated RSD facet. Researchers then discussed affective descriptors as a group, in order to refine and reach agreement upon these inductively-derived categories. The frequency count of statements that connected to each descriptor is presented in Table 2 (in the next section). This quantification is merely intended to provide a sense of how commonly the participants portrayed the facets in terms of particular affective descriptors; generalisations cannot be derived from this data. Quantification is common in qualitative research [38] in order to provide a sense of emphasis among those interviewed rather than a sense of representativeness. Quotes that epitomise graduate statements for each descriptor are provided to give a sense of how graduates talked about the affective aspects of each facet. All italics in the quotes from interviews were added by the authors. This use of the RSD facets for organising the data as an a priori framework enabled a comparison of, and contrast of published affective descriptors in the RSD that were derived from the academic community [19] and the graduates’ sense of affect associated with research and EBP in clinical employment.

## Results

The results are in two sections. The first section addresses Question 1 and considers graduates’ retrospective look at the development of skills when they were previously students in university, and where they attribute this development to specific aspects of the BOH. The second section addresses Question 2 and is the graduates’ current perspective on their use of research skills and EBP in clinical employment, with the analytical focus on the affective aspects of the data, in keeping with the research question. Findings of question 2, comprising affective descriptors, are organised according to the RSD facets**.**

### Graduates’ perspectives of the development of research skills and EBP in the BOH

The first research question concerned the extent to which the graduates retrospectively attributed the development of their research skills to the explicit, designed features of the BOH.

#### Scaffolding of research process learning

One graduate articulated the designed processes of the BOH curriculum in terms of explicit research skill development:

“Well, I guess there’s always some sense of trepidation... they were scaling up from assisting us and then giving us more autonomy as we went.” (Graduate D).

This conveys the perception that scaffolding towards autonomy was, for this graduate, a recognizable design feature of the program, from lecturer-directed learning towards student instigation. Another graduate, who had also tutored in the program, noted the time-on-task needed to make the research process ingrained for other students, spanning multiple semesters:

“… towards the end of the degree, I saw that they started to get it. It just sort of clicked to them. But initially it was really hard, and also their academic writing, they just didn’t understand.” (Graduate I).

According to this graduate, it was not until the last two semesters that many students came to understand the research process being explicitly scaffolded throughout the degree using the RSD. While from the academics’ perspective the research skill development process was made explicit early in the program to students, this was not always evident to graduates when they were reflecting back on their experiences as students:

“I think maybe one of the biggest things would be making students *more aware* of the research skills when they’re doing the assignments, so things like the [First Semester] General Studies course and that sort of thing, that is teaching them the skills and why you do it, and that kind of thing.” (Graduate F).

In addition to recommendations on how to make research skill development more explicit, graduates made suggestions on the relevance of the research they had carried out at university. Only one graduate reported a clear sense of awareness during the degree that the research could be used in the workplace in the future:

“…our topic was quite relevant to what we do. So I actually enjoyed researching because I could apply it to what I actually do in the clinic.” (Graduate E).

Graduates rarely articulated this sense of relevance and potential for longer-term application when considering, retrospectively, research they conducted *while* at university. One third of graduates interviewed (B, F and H) explicitly stated that they were not conscious of the relevance of research skills and EBP until they started working. One of them noted:

“At some stages of the course things that we learned, I didn’t understand the relevance of why we were learning it, such as evidence-based dentistry, just different little things. But now that I’m out practicing, it’s amazing how much those skills have come into practice, and at the time it wasn’t really explained to me in a way that I would need this later on, or you know, it was just expected that it was part of the curriculum. So, I really didn’t like evidence-based dentistry. That was probably my least favorite subject during the course. However, now, I use that so much.” (Graduate H).

Another graduate recommended:

“…they did try to make us use the knowledge and apply it to the clinical environment…but maybe that’s something that could have been *focused on a little bit more*.…unless you have somebody or a moment where they tell you that you need to apply it, or even if we were in the clinical situation and your tutors were able to say to you, this is like what we did in this assignment, this is where we’re using this.” (Graduate F).

Without this sense of relevance, some graduates found the process could be demotivating:

“… I’m more than happy to come and talk to them [current students], especially the ones thinking: why are we doing this? Because I was the same. I sat there, and I was, like, Oh, this is really boring, looking at statistics.” (Graduate H).

‘Looking at statistics’ for many is not a search for trends and insights, but a complex encounter with data that is a mysterious and demotivating process.

There was broad support from all graduates interviewed that this long-term learning, often accompanied by strong negative emotions such as ‘trepidation’ or ‘boring’, was worth it in the long run, and required a learning process that slowly built research skills and knowledge:

“You have to research it, you don’t get fed stuff anymore. You have to go, research it, sit down, analyse what’s important and what’s not. So yes, it slowly did lead up to a better research in third year. I think if we started researching in third year, we wouldn’t produce a high quality piece of work at all.” (Graduate B).

The phrase ‘you don’t get fed stuff anymore’ connoted the need to take ownership of learning through research processes and the sense that students are expected to ‘feed’ themselves. The retrospective knowledge that the BOH did not wait until the third year of the degree to develop these skills, but rather started this process from the beginning provided this graduate with the sense of capacity to engage in research processes and EBP in a higher quality way. With the above suggestions in the foreground, one graduate noted how the degree prepared her for work in clinics:

“You’re not going to be babied in a workforce, so you’ve got to do things yourself, and you’ve got to think proactive. I think in a sense that was a *little bit of an underlying message while you’re there, to be proactive in your own research* and not be babied all the time, because that’s what it gets down to.” (Graduate C).

‘Proactive in your own research’ equates to a retrospective understanding that in the employment context, working with patients and colleagues, one has to drive things. This was true for participants’ university research, and it carried into research and EBP in their clinical employment. Looking at the process of explicit cognitive research skill development from first year to employment, one graduate articulated the ‘big picture’ as follows:

“… it encourages all its graduates to have a *mindset of research* on focused learning, lifelong learning and to know that study doesn’t stop at the end of the course…” (Graduate D).

All the interviewed graduates recognised, retrospectively, that the BOH had explicitly and incrementally developed their research skills and EBP. This was the mindset that the graduates brought to employment, and is suggestive of a raft of skills associated with research and EBP. However, were graduates able to effectively move from knowledge and skills of the mind, to connected behaviours, values and attitudes in clinics? What were their affective descriptions of using research skills and EBP with patients when employed?

### Affective dimensions of research and EBP at work, organised by the six RSD facets

Table 2 shows the affective descriptors associated with each RSD facet, based on the coding of graduates’ interview responses.

**Table 2:** Affective Descriptors emerging from the interviews with nine students, organised by RSD Facet

	RSD Facet	Affective Descriptor	Attributing Student	Number of Students (***n*** = 9)
1	Embark & Clarify	Curious	All	9
		Passionate	A, B, G, H	4
		Owning (the process)	B	1
		Interested (in learning)	F	1
		Dissatisfied (with status quo)	I	1
		Bored	B	1
2	Find and Generate	Determined	A, B, D, E, F, G, H, I	8
		Re-embark: Horrible to find	A	1
3	Evaluate & Reflect	Discerning	C, D, E, H, I, B	6
		Re-embark: Self-identified knowledge gap	G	1
4	Organise & Manage	Harmonising	A, B, C, E, G, H	6
5	Analyse & Synthesise	Creative	A, B, D, E, G, H, I	7
		Re-embarking: Justify decisions	D	1
6	Communicate & Apply	Constructive	All	9
		Re-embarking: Patient instigation	A, C, D, E, F, H	6

For all the interviewed graduates, there was a strong and close connection between the research skills learned at university, and how they thought as clinicians with patients at the time of the interview:

“So the way that you have to think about it when you *research* is that the first thing you would think when you would see them [patients] as a clinician. They [patients] provide you with a brief summary, and you have to think about their background. First off, that’s how you *think as a clinician* – the background, all their history, so socially, dentally, medically.” (Graduate G).

Research Question 2 probed the attitudes and values that lead to sustained behaviours around EBP. The following affective-oriented data, addressing Research Question 2, is organised according to the six facets of the RSD.

#### Embark and clarify

Beginning research is a complex endeavour, and graduates of the BOH identified multiple affective factors connected to the instigation of research and EBP. Six of these were identified under the category of *Embark and Clarify* as ‘kicking off factors’, i.e., factors that instigate or drive the research from the beginning. There were also four ‘re-embarking’ descriptors, which are those that drove the research into another cycle, each one associated with one of four of the other five facets, as shown in Table 2. Together, this provides a total of ten affective factors associated with *Embark and Clarify* that were identified from these interviews, many more than the affective descriptors *curious* and *empathetic* in in the RSD [16]. The six affective descriptors associated with *Embark and Clarify* as ‘kicking-off’ factors that drove graduate EBP are:

##### Curious

All nine graduates mentioned this affective descriptor during their interviews, and it was a major motivating factor to impel research and EBP. One student stated:

“I think difficult situations, situations you’ve never seen before; clients with rare conditions or something that you’re just *curious* about, that’s when you need to do some research.” (Graduate E).

Being curious may be triggered unexpectedly, or it may be endemic to a graduate’s interests, such as curiosity about rare conditions.

##### Passion

While similar to ‘curious’ in its positive impetus, four graduates explicitly used the word ‘passion/ate’ in ways that connected to *Embark and Clarify*:

“I was like really into it and I was researching all this stuff...like you’ve got to find something that really excites you and intrigues you ... and being *passionate* about a topic...” (Graduate A).

Intrigue is synonymous with curiosity, but passion connotes the sense of a cause to pursue, almost a missionary zeal to bring about the object of desire, such as public health benefits from good oral hygiene. Whereas one isn’t sure where curiosity will lead, passion is often directed towards some big picture.

##### Owning the process

There was one graduate who discussed ownership as an important affective element:

“Yes, I like to motivate my patients, because I think it’s quite important. You can’t get the motivation if you just pump advice into them. … That’s *my own kind of project,* where I want to see where this can take me with patients and see where it can start changing.” (Graduate B).

The form this graduate’s research and EBP took was initiating her own projects that made a difference to patients, because she wanted to ‘see where this can take me’ in her learning about what real positive change she could personally bring about with patients.

##### Interested (in learning)

One graduate articulated a sustained interest in learning as a driver of research and EBP:

“I guess I just realised how it’s quite good to find new information and to really explore specific areas, but I think it’s just the interest in that and *to continue to learn* and develop, that kind of thing, is the biggest thing that’s kind of inspiring me to do it.” (Graduate F).

Here, the source of inspiration for this graduate to embark was interest in learning as a human endeavour, akin to life-long learning.

##### Dissatisfied (with the status quo)

One graduate disparaged the idea of not researching, suggesting that some people would stick with their increasingly outdated ways, and so the negative implications of not researching were a reason for this graduate to continue to learn:

“But I actually think in private practice a lot of people are *less motivated* to actually just go and look something up or whatever online or things like that*.* They just *sort of stick* with their ways.” **(**Graduate I).

##### Bored

One aspect of working life for a graduate was an unexpected driver of engaging in research:

“… if I get bored, or if I find my work being too monotonous, I might end up researching.” (Graduate B).

For this graduate, there was a sense that research provided a useful, helpful, or at least distracting, alternative to tedium.

Surprisingly, these six affective drivers towards the instigation of learning were not the only affective factors identified for this facet. As noted previously, in four of the five facets below, there are affective descriptors associated with a ‘re-embarking’ process. These descriptors point to situations where graduates were engaged in some other facet of research, but then identified affective drivers that motivated them to re-embark and re-clarify their learning processes.

#### Find and generate

##### Determined

Eight graduates made statements pertaining to finding information or generating data that were in keeping with the existing RSD affective descriptor of *determined*. One of the graduates said that the areas she looked into:

“… were really interesting so I just wanted to know more so I just *kept on looking* into it.” (Graduate A).

Frequently, there was a clear articulation of the intersection between what caused each graduate to start and the desire to continue. For Graduate A, the driver was around interest in the topic. For others, the driver was concern for a good patient outcome. But for eight of the nine graduates, there was, in determination, the sense of a need for due diligence, not settling for taking whatever information came to hand but to:

“… build up research, so whatever you found on the first day may have not been included in your end product.” (Graduate G).

**Fig. 2:**
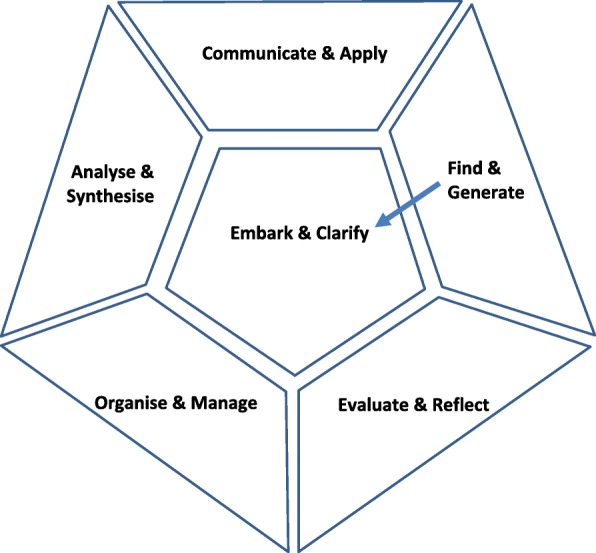
a connection between *Find & generate* and re-*Embark & clarify*

Whereas descriptors of *embarking* on research were numerous in the interview transcripts, those for Facet B (*Find and Generate*) were characterised primarily by determination; a determined graduate who keeps going, keeps looking, keeps doing more to find optimum information. There was, in addition, a re-embarking aspect to *Find and Generate* (see Fig. 2), where during the process of finding information, the need to once again seek clarity of purpose becomes paramount.

Re-embarking comprises the common situation where a student may be focussing on a certain facet of researching and EBP, such as finding and generating, but aspects of the process take the student back to the need to clarify direction and possibly restate patient need, research question, problem definition, etc. Find and Generate, and the next three facets, provide evidence of students engaging in one facet and then ‘returning to the centre’ (see Fig. 2) and re-embarking from there. Re-embarking is symptomatic of the fluidity of the RSD, the porous nature of the delineations of facets and of the non-sequential, non-predictable use of skills in sophisticated contexts that require research skill and EBP.

##### *Re-embarking:* horrible to find

The aspect of *Find and Generate* that became a driver of the research process was most clearly represented by a graduate reflecting back on a university assessment task, as this was not mentioned for the work context by those interviewed:

“We chose a horrible topic – like very interesting for us but absolutely horrible to find any information on research was just like down the toilet. We had to like…broaden it so much that we could get like…the info we needed. It was absolutely ridiculous.” (Graduate A).

This shows the clear inter-relationship between *finding* and *embarking*, the mutual interdependence, and the non-linear, recursive movements between facets that are typical of research and EBP. While a topic was chosen by this interviewee’s student group more than one  year previously, the realities of a topic initially deemed ‘very interesting’ led these students ‘down the toilet’ of a lack of information. *Broadening* is a re-embarking and re-clarifying process. In positive circumstances, this could become a great learning exercise for students, showing, for example, the need to take a broad look before plunging too deep. In more negative instances like that above, it could be considered ‘absolutely ridiculous’, frustrating, or even inhibitory to learning.

#### Evaluate and reflect

The facet *Evaluate and Reflect* was a daily reality, with tangible clinical consequences for patients and dental colleagues, and affective comments were mainly in keeping with the existing RSD affective descriptor of *discerning*. For those interviewed, it had a strong affective side.

##### Discerning

Six graduates mentioned affective aspects that were categorised as “discerning”, with one particularly evocative insight into this affective aspect of work:

“I asked ‘Why are you doing that?’ and she [dentist] would be like ‘Well why are you doing that?’ and it would be like ‘I was taught to do this because this is holistic; it gives less radiation, blah, blah, blah’ and she would be like ‘Well I need to do this because it’s more accurate, it’s closer to the teeth’ so there’s reasons on both sides but sometimes it would be very tense because we’d be like ‘Well that’s all right, I wasn’t taught that’ so we do have to research and compare and then it’s just a matter of choice ultimately.” (Graduate A).

This clinic-based realisation that there are different justifications - ‘reasons on both sides’ - for dealing with the same situation led to ‘very tense’ feelings and decisions that seemed ‘a matter of choice’. This realisation is a major aspect of the search for evidence in practice, with different evidence bases that may be called on, and with differing rationale. EBP requires the discernment that enables a moving forward when differences of opinion threaten to bog down the whole process.

In terms of the drive to be effectively discerning, one graduate provided insight into being discerning about products:

“Definitely new products in the clinic could be one where it’s new, you don’t know what it is, and then you have an inquiry, *you want* to find out more about it, and then *you want to evaluate* all the information you have on it. So if they’ve got any clinical studies that prove that the product works well and that it is good, then you do *want to have a look* into it. Was it a randomly controlled trial; was it a non-biased trial; was it privately investigated or was it looked at by a lot of different people? … Do you want to be promoting this product to people that are going to be using it for *the rest of their life*?” (Graduate G).

Here, evaluation is perceived to be vital, because of the long-term impact of sub-optimum products on patients and here the graduate *wants* to look into it, has a *desire* to be evaluative. This is evidence of the top of the Affective Domain taxonomy, internalised as *discerning*. The graduate’s values around patient care extended way beyond the immediate concerns of the clinic to the rest of a patient’s life. Another expression of the need for discernment was more negative:

“I was very surprised at the amount of people who didn’t know how to research properly, and that is a bit scary in a way, and these are academic students who don’t know how to research properly, so imagine people just pulling information from anywhere and just don’t know where it’s come from and use it incorrectly.” (Graduate I).

##### *Re-embarking:* self-identified knowledge gap

The *Evaluate and Reflect* facet had a descriptor that was a re-embarking factor for research, in terms of a self-identified knowledge gap:

“So, it’s basically when you don’t know, when you’re not confident in doing what you’re meant to do in a safe way, you should do some research.” (Graduate E).

This is a hallmark of a willingness to learn, understanding the limits of one’s own knowledge. Reflection on current knowledge in the clinical context led to self-realised deficits in knowledge, and so re-instigated fresh research processes by the graduate: ‘when you don’t know- you should do some research’*.* This self-discernment which recognises knowledge shortfalls is a hallmark of professionals, who know when to make decisions, when to seek further information, and when to refer patients on to other practitioners who have the necessary knowledge.

#### Organise and manage

The seemingly mundane aspects of research in the *Organise & Manage* facet were addressed by seven graduates and were in keeping with the RSD affective descriptor of *harmonising*.

##### Harmonising

One graduate provided a story of passionate controversy over how and when to chart aspects of patient bone loss, involving both the organisation of patient data and management of interactions in a team of two.

“Yeah, one of the things – it seems so pathetic and minor, but it’s about periodontal charting, so basically the measurements around each of the teeth which indicate if there’s been any bone loss or inflammation… if she saw the patient before I did, she would do the chart and then I would get the patient whereas usually as an oral health therapist I would do my own chart and I would just go from my chart every time I see the patient. But she is interested in Perio so she does a chart, so there was already a bit of a kafuffle with that because I am just like ‘Ugh, this is my job...’ so it was very stressful, but we got through that by saying like ‘Okay, so the hygienist - it’s more time effective if the hygienist does it.’” (Graduate A).

The charts themselves were important organisational structures that provided an understanding of what was happening to the patient over an extended timeframe and was an example of vital clinician-generated data for EBP. The hygienist and the dentist were both professionally interested in charting and the practical use of the information to ‘just go from [the] chart every time [they saw] the patient’. This led to a team management ‘kafuffle’ regarding who was responsible for the organisation of patient data. In this case, the solution was to demarcate roles through collaborative consultation, with the eventual decision that it would be ‘time effective’ for the hygienist to take charge of charting. This sense of initial discord and the need to find a way forward is in keeping with the development of harmonious relationships, just as the helpful tracking of patient data provides a sense of harmony of information. Graduates found that, in general, organisational processes were enabling, not ends in themselves. The affective aspects of team management that seemed ‘so pathetic and minor’ were as pivotal to long-term patient wellbeing as were the organisational charts. There were no explicit recounts in the interview transcripts of instances of *Organise and Manage* leading to re-embarking, though it is quite possible that this occurs.

#### Analysis and synthesis

Seven students provided statements that related to *analyse & synthesise* and conveyed the descriptor “creative”.

##### Creative

One student indicated the pertinence of her research skills, when she explored a product that was intentionally excluded from the BOH content when volunteering after leaving university:

“Before I left for Cambodia, I actually took a silver fluoride which is a product that we didn’t even actually come into contact with in the Bachelor of Oral Health… because it stains teeth and there’s all these things that they don’t like aesthetically so that’s why we weren’t using it. But it’s like an amazing product because I thought that might be really beneficial for Cambodia because they don’t get care often and they’re considered more rural… I ended up purchasing some [silver fluoride] and taking it over with me and I was using it a lot when I was over there.” (Graduate A).

While this quote addresses multiple facets of research, here it is the decision to buy and take silver fluoride that is impressive - an analysis and final synthesis of the chosen solution as the culmination of her complex research and EBP processes. In the affective domain, this required creativity, thinking outside the box, even outside of BOH course content.

##### Re-embarking: justification of decisions

Analysis and synthesis were applied on a daily basis in regular clinical environments, and sometimes became re-embarking factors in the research process:

“…one patient I believe whenever I give an amalgam restoration, I need to *justify* my decision and I *explain* the pros and cons of the material.” (Graduate D).

The need to justify and explain, synthesising information and using analysis to support decisions, drove the research process in which the graduate subsequently engaged. Without this patient pressure, the graduate would have carried out another standard amalgam restoration, following a standard procedure; the need to justify to the patient re-launched the graduate into research and EBP with subsequent reporting to the patient.

#### Communicate and apply

The current RSD affective facet ‘constructive’ that corresponds to *Communicate and Apply*, as noted earlier, provides a sense of the value and attitude to ‘build up’, as opposed to a desire to persuade.

##### Constructive

Statements about the application of procedures and products that were suited to each patient were made by all graduates. Graduates’ statements around *Communicate and Apply* (Facet F) provide a sense of desire to do the best for a patient and to be constructive:

“Because when I’m doing research and looking things up, it is to *apply* to my patients and to apply to the clinical setting, so I can’t just go on to Wikipedia and find something and say, well, that must be the answer. You’ve really got to analyse it and say, is *this what’s going to work in practice, and is this what’s best practice for my patient*?” (Graduate F).

##### Re-embarking: patient instigation

Strikingly, six graduates stated research that that was instigated when patients asked questions or sought clarification, as one graduate noted:

*“*…the patients who ask me questions about links between a heart problem and a periodontal disease…you think, well, the uni taught me well enough to know about the studies that are out there, and I can look them up and I can talk to my patients about research and things like that. So yes, it’s just *amazing.*” (Graduate C).

Strong interactions with patients were indicated more frequently than any other facet in interviews as leading to re-embarkation on research processes. Students commonly found themselves re-embarking on research when patients sought further clarification about treatments:

“…I can think of at the moment was a particular patient who was against radiation, so against dental x-rays, and obviously having to – I mean, I could give her a general description of why we do it and what I’m recommending it for. But I then researched more into it and found some evidence base into the things she was concerned about…So I guess that’s a positive influence that came from my research...because she could see the evidence base behind what we were doing and why I was wanting to take the x-rays. It kind of alleviated her concerns a bit about the exposure to radiation and that sort of thing.” (Graduate F).

Communication with patients thus drove graduates towards research processes which were accompanied by a strong desire to be *constructive*, to gain not only positive physical health outcomes, but also positive affective outcomes for patients and to alleviate concerns.

## Discussion

Graduates’ attributed the development of skills associated with researching and EBP to the BOH program, addressing Research Question 1 explicitly. There was a clear sense from the nine graduates interviewed that the program developed the knowledge and research skills needed, and that there was, retrospectively, a well-scaffolded process across the years to achieve this. However, this process was rarely evident to them at the time of university study, with only one graduate articulating that he had a sense of skill development while in the program.

Three graduates explicitly stated they only realised in hindsight about the ways that the program developed their research skills and suggested ways to make the process itself more obvious to students while they were studying, instead of it dawning on them at the ‘end of the degree… [when] it just sort of clicked’.They also requested ways to heighten the sense of the value of these skills so students could perceive, prospectively, the value of the skills for employment. Participating graduates desired that the BOH be engineered to provide more practical and affective reasons for engagement in the development of skills associated with research and EBP, using terms such as ‘boring’ and ‘trepidation’, but also ‘amazing’ when graduates realized there was a point and that the skill development ‘slowly did lead up to a better research in third year’ and was relevant to employed practice. Overall, the nine employed graduates who were interviewed attributed the development of research skills and EBP to the BOH entire program, in keeping with studies that have shown that, not only is student EBP and research skill development relatively slow and incremental [21, 39], but that students themselves have called for the slow, scaffolded buildup of the associated skills [40].

However, what was the interviewed Oral Health graduates’ perspective of these skills and practices in employment?

### Affective drivers of graduates’ use of research skill and EBP

Research Question 2 was addressed by graduates discussing their use, in employment contexts 1 year after graduation, of the research skills and EBP developed in the BOH. The results focussed on the affective descriptors that lined up with the six RSD facets. In these nine interviews there were six affective descriptors for *Embark and Clarify* (Facet A) which form a starting point of purpose and intentionality, and two descriptors for each of the other facets, except *Organise and Manage* (Facet D), which had one. However, for the other facets, the second affective descriptor was primarily a way to ‘re-embark’, i.e., a point from which to begin another round of researching and EBP.

The diversity of descriptors associated with *Embark & Clarify* illustrates numerous impelling factors for research and EBP and is perhaps indicative of the complexities of starting, of restarting when momentum is lost, or of ongoing fine-tuning and clarifying direction and purpose. This complexity associated with *Embark and Clarify*, and the need to be clear about direction taken, suggests that this facet may be pivotal for the transfer of the knowledge and skills developed at university to the values, attitudes and behaviours associated with EBP for patient care. Drivers of embarkation included the following: *curious, passionate, ownership, interest in learning, dissatisfied with status quo* and *bored.* Drivers to re-embark and re-clarify included such aspects as information that was *horrible to find, self-identified knowledge gaps*, *justification of decisions* and *patient instigation*. One study found that curiosity enabled allied health students to engage in EBP processes and related this engagement to positive personal attitudes towards the subject [41]. However, graduates in this current study were impelled to apply EBP by factors, especially patient-related factors, that were at times more pressing than curiosity.

The current published RSD descriptor of *curious* was then merely one of the drivers named by all the graduates interviewed, but together with the other nine descriptors, need to be considered as a potentially important set. The diversity of descriptors for *Embark & Clarify* suggest that actual working contexts comprise many affective elements that imbue EBP with a sense of purpose and drive graduates to apply their knowledge and skills to patient care. This research addresses the gaps identified by quantitative research that called for the identification of factors that drive the transfer of knowledge and skills into attitudes and behaviours [2]. This current qualitative research does not reveal if the above descriptors comprise a partial or complete set, or how representative each descriptor is, but it does provide a deep and rich qualitative exploration starting point for future quantitative research. However, additional qualitative research is needed first to further probe the affective aspects that initiate EBP in working environments.

The other facets of the RSD, when interview data was thematically analysed, returned affective descriptors in keeping with existing published ones. Considering the majority of graduates who were not interviewed, and graduates from other programs, there may be greater diversity of descriptors for these facets, and further research is needed to determine in order to capture broad, diverse affective descriptors, facet by facet, that reflect graduates’ experiences. For example, Sonographer educators have indicated, for *Find & Generate*, that *meticulous* is more pertinent in ultrasound provision to pregnant mothers than *determined* [42].

By the time graduates in this study were interviewed, they had internalised values and attitudes associated with research and EBP as shown by their self-reported behaviours, and there are four possibilities at least for the development of this top, *characterisation* level, of Bloom’s affective taxonomy [15]. The first possibility, of course, is that some or all of those interviewed misrepresented or inflated the accounts of behaviours with patients. They may have selected out the stories that were rich in values and attitudes, and expressed as exemplary patient-oriented EBP. However, the use of semi-structured interviews to gain graduates actual experiences does provide data that is more credible than a number of other studies of the affective domain. Explicit and hard-set delineations of affective domain in self-report surveys, for example, were used when assessing student affect in some studies, and this can be counter-productive unless sensitively managed [19]. Quantitative instruments designed to capture affective aspects may not be sufficiently sensitive to capture the nuances of the affective domain [23].

A second possibility is that some commenced the BOH program with these attitudes and values already well-developed and internalised.

A third possibility is that some graduates developed these attitudes and values at the level of *characterisation* during the degree. The BOH use of the RSD across multiple semesters of the program itself is a strong commitment to, and modelling of, the importance of continuous learning. This idea is supported by a review of studies which found that empathy development needed the extended timeframe of a whole degree to be effectively nurtured [14].

A fourth possibility is that values and attitudes associated with EBP were modelled and promoted in the BOH program of study, and some students operated during the degree at the Bloom’s Affective taxonomy levels of *receive or respond* but not yet at the level of ‘organisation’ or characterisation’. One study [43] found that students may not be able to demonstrate affective behavior at their work place if there is no empathy evident or elicited. From this perspective, empathy is essential, a precondition for the development of affective skills in health professionals’ personal as well as professional growth. Values and attitudes associated with EBP were, for the interviewed employed BOH graduates, deeply internalised and there is a sense that having patients under their duty of care provided a more affective impact than when they had previously seen patients in university-organised clinics. Linking what is learnt in the classroom with the practice in the work place is seen to be a vital factor in the development of attitudes, values and behaviours [44]. That is, these values may have become increasingly internalised as a result of patient encounters until employed graduates ultimately expressed these values at the level of characterization during EBP.

More than values and attitudes, however, are needed to work beyond the level of Bloom’s *valuing*; to work at the level of *characterisation* requires a knowledge and skill base for the values to be manifest as sustained and appropriate behaviours. The graduates indicated that they were, in general, less aware of the knowledge and skills of researching and EBP early in the degree and became more aware by the end of the degree, and it is likely that the values and attitudes at the characterisation level emerged later than the knowledge and skill base they are connected to. Therefore, the second and third possibilities above provide less impetus, are less enabling and seem less likely to equip students with values, attitudes and sustained behaviours for EBP, than when students work directly with their own patients.

In summary, it is possible that some students of the BOH may have enrolled in the program with pre-existing values and attitudes associated with EBP, or developed and manifested them during the degree, but more likely the skills and attitudes were developed but latent until employed with patients under their duty of care. What is vital to consider is how to provide opportunity and motivation to enable student or graduate attitudes and values to work at the characterisation level in ways that are fruitful and not counterproductive.

The factors that instigate research processes in the working context are diverse, and they are the factors that seem to prompt or nurture internalised values and attitudes of researching and EBP for those interviewed. Curricula that focus on promotion of facts and recall, or provide little opportunity for students to learn from mistakes, are more likely to prompt students to rely on teacher-directed learning and less likely to encourage values associated with patient-oriented EBP. The results of this study suggest that the working context provides natural incentives to engage affectively in EBP, incentives that are less tangible to students in the context of university study. For example, graduates in working contexts are exposed to patient requests and demands, which elicit spontaneously affective responses. However, elicitation of affective elements in clinics may well be contingent on whether graduates have sufficiently developed cognitive skills and knowledge for research and EBP. In the interviews it seems that the affective dimensions of work acted in concert with, and were mutually supportive of, the cognitive skills associated with researching and EBP. The graduates, it seems, had already developed needed knowledge and skills at university, and when these skills and knowledge were brought to bear case by case as affectively elicited by patients, this aided the transfer of research skills to EBP that enabled patient care.

Results from the current study, then, concur with the assertion that teaching EBP is more than simply providing students with knowledge about research and evidence, but must consider how best to foster values and attitudes [45]. This may include strengthening the skills for teaching the affective domain as a focus of health science programs [46]. However, the difficulty with imparting and assessing in-program values and attitudes in an effective way needs further attention, because design intentions around affect are not guaranteed to be realised [12]. There are risks associated with telling students about attitudes they should have in an explicit teaching mode, where the latter fosters a compliance mentality that does not guarantee transfer to work. A push to teach values and attitudes explicitly should be tempered with the possibility, seen in this study, that proximity to patients and their needs elicit strong and spontaneously positive student responses if the students are equipped with relevant knowledge and skills. The findings from this current study may be a result of workplace environments being more varied and complex than those in university, and therefore demand the application of a greater number of skills, both cognitive and affective [47], a point supported by a study of postgraduates’ learning in GP training and of the development of compassion during medical intern year [48]. This current study, while non-generalisable, provides detail that shows the richness of affective engagement in working environments in terms of the six facets of the RSD, engagement that seems to prompt transfer of knowledge and skills to EBP.

### Contrasting affective engagement at university and in employment

The above analysis and discussion provide a picture of surprising contrast between affective engagement in university research and EBP when employed. A qualitative representation of this difference, based on the graduates’ interviews, is presented in Table 3. The contrast of university study with employment suggests that study is *affectively* more difficult, because the natural incentives associated with patient care are significantly less at university. Student questions around purpose and pointlessness of research skills and EBP tended to evaporate for employed graduates dealing with patients.

**Table 3:** affective engagement in research and EBP during university studies vs employment

	Affective engagement in university-based research & EBP	Affective engagement in employment-based research & EBP
1	Lower perceived relevance	High perceived relevance
2	High frustration expressed	No frustration expressed
3	High uncertainty about purpose	High certainty of purpose
4	A range of autonomy evident in interview data, but problems evident for high autonomy	High level of autonomy/self-regulation evident in interview data

If the attitudes associated with research and EBP do not ‘characterise’ the students while they complete their study or on graduation, is there still time for these attitudes and values to emerge? This is a practical question, because an explicit teaching emphasis of attitudes and values [49] is not a guarantee that students will form these attitudes and values in a program of study [50]. Moreover, the affective and compelling nature of engagement with one’s own patients in employment may be needed for many students-come-graduates to externalise their values as behaviours. The sense of professionalism and duty of care when dealing with one’s own patients is a stronger motivator towards attitudes and sustained behaviours for EBP when compared to dealing with patients in clinical contexts that are university-supervised. It may be this emotive connection with one’s own patients enabled and drove graduates’ appropriate, professional, caring and informed responses to patients’ needs.

In this study, without structured elicitation of affective considerations in the interviews, the graduates quite naturally related their cognitive learning of research skills and EBP in their degree to their highly affective clinical working contexts, one year after completion of their degree. The employment enterprise was an emotional experience for them, patient by patient, full of curiosity, passion and care. The affective elements of their research skills were in the fore, where these graduates determined their own learning needs and those of their patients.

### Recommendations

We recommend that similar fine-grained research be conducted in a number of institutions and contexts that develop research skills and EBP in order to look for similarities and differences with this present study. This may provide a strong basis to develop constructs that enable quantification of the complex interplay of cognitive and affective facets that comprise researching and EBP.

Further research should consider the long-term outcomes of health science programs concerning the learning of graduates with measures that are sufficiently sensitive to affective dimensions. This should include studies on the outcomes of RSD framework when used to conceptualise how to scaffold the skills associated with research and EBP across degree programs in numerous and diverse contexts. In addition, the RSD’s cognitive and affective facets may be used as an analytical framework to explore the dimensions of research and EBP during and after whole degree programs.

### Limitations

This qualitative study sought to uncover the richness of experience of employed graduates and the nine participants were a small proportion of all graduates of the BOH. As such, their cognitive and affective experiences are not broadly representative of graduates from the BOH program or any other program. However, because it is harder to capture detailed perspectives about a degree from long-term graduates than from students or recent graduates, this data on long-term outcomes of a specific intervention is hard-won and provides valuable insights. The data collected is valuable also because the graduates attribute the development of skills to specific aspects of the BOH program, and because it is rich in descriptive detail and provides fundamental insights into long-term affective outcomes of research skill development.

With only a small proportion of each cohort interviewed, there is a potential selection bias, where only those who found the RSD process beneficial turned up for an interview. For example, only graduates who were employed attended interviews. In addition, as noted earlier, a positive confirmation bias may have been prevalent among the participants, where they wanted to affirm their perception of what the interviewer wanted to hear. To partially overcome these potential biases, disconfirming evince was sought, and graduates did commonly articulate more negative aspects of the program, such as ‘boring’, suggesting confirmation bias was not a strong factor.

## Conclusion

This rich qualitative research made a small but valuable contribution towards filling the research gap, identified by quantitative researchers, in understanding the processes of transfer of knowledge and skills to attitudes, values and sustained behaviours in clinic-based employment. The findings of this research are meant to provide deep understanding of the transfer as it was experienced by those interviewed and are not in any way generalisable. However, the conceptual framework and methodology employed may be used more broadly to continue to address the transfer gap.

From the perspective of the interviewed employed graduates of the Bachelor of Oral Health the cognitive skills of research and IBP were already in place on graduation. However, their subsequent move into employment and engagement with patients, seem to have been a major factor that enabled their values and attitudes associated with research and EBP to operate at the top level of Bloom’s Affective domain of *characterization*. That is their knowledge and skills were operationalised by affective elements to provoke appropriate patient-oriented behaviours. Primed as they were by the BOH’s explicit knowledge and research skill development, interviewed graduates’ interactions with patients in particular prompted the affective and effective transfer of their knowledge and skills to their evidence based practice in clinics.

The published affective descriptors of the Research Skill Development framework reflected much of the affective experience of interviewed graduates, and this was the case for *determined, discerning, harmonising, creative and constructive*. However, affective descriptors for the facet *Embark and Clarify* were much more diverse, rich and complex than the published ones, *curious* and *empathetic* [19]. There were nine other affective descriptors for *embark and clarify,* in addition to *curious* from these interviews, including *passionate, owning the process, interest in learning, dissatisfaction with the status quo, bored*, information that was *horrible to find, self-identified knowledge gaps*, the *need to justify decisions* and *patient instigation*. Together, these show that embarking on and clarifying learning through research and EBP is complex and multi-factored, especially when driven by patient care. These descriptors provide deep and rich understanding of affective elements that drive the transfer of graduates’ knowledge and skills from their degrees into attitudes, values and behaviours of evidence based practice when they are employed in clinics.

## Data Availability

The datasets generated and analysed during the current study are not publicly available because of ethics committee requirement to protect the anonymity of participants.

## Appendix

*Interview Questions for Semi-Structured Interviews with Oral Health Graduates conducted 2011–2013.*

When you look back at your university studies, what stands out one year later?

What is your current employment situation? How long have you been working there?

Over the past year, have you needed to use any research skills in your current job?

Can you suggest situations where you have used research skills. Did this have an impact in your workplace?

Has your perception of research changed since you’ve graduated?

The RSD assessment tasks were examples where they focused on explicitly developing your research skills. What do you think was the point of those assignments?

Were you aware that the intention behind these assignments was to help you develop research skills?

In the third year, you had less structure and more open-ended inquiry research project. What was your experience of that project?

Did the first and second year research skill assignments help prepare you for the research project in third year?

What do you think were the kinds of research skills being taught in the Bachelor of Oral Health?

Did you have that awareness research skills being taught while you were studying?

You mentioned a couple of skills that the Bachelor of oral Health was developing. Are there any similar skills that you need to use now in the workplace?

Do you think your employer values these research skills?

What came across as a surprise, in a positive or a negative way, after you started working?

As you might know, the Bachelor of Oral Health focuses a lot on building research skill. Do you think this approach needs to be changed; if so, what to? If not, can you suggest any improvements to how they could do this better?

Did the teaching of research skills in Bachelor of Oral Health motivate you in your study, while you were studying?

## Abbreviations

BOH: Bachelor of Oral Health
EBP: Evidence Based Practice
RSD: Research Skill Development
